# ACVIM consensus statement guidelines for the classification, diagnosis, and management of cardiomyopathies in cats

**DOI:** 10.1111/jvim.15745

**Published:** 2020-04-03

**Authors:** Virginia Luis Fuentes, Jonathan Abbott, Valérie Chetboul, Etienne Côté, Philip R. Fox, Jens Häggström, Mark D. Kittleson, Karsten Schober, Joshua A. Stern

**Affiliations:** ^1^ Department of Clinical Science and Services Royal Veterinary College Hatfield United Kingdom; ^2^ Department of Small Animal Clinical Sciences, College of Veterinary Medicine University of Tennessee Knoxville Tennessee USA; ^3^ Alfort Cardiology Unit (UCA), Université Paris‐Est, École Nationale Vétérinaire d'Alfort Centre Hospitalier Universitaire Vétérinaire d'Alfort (CHUVA) Maisons‐Alfort cedex France; ^4^ Department of Companion Animals, Atlantic Veterinary College University of Prince Edward Island Charlottetown Prince Edward Island Canada; ^5^ Animal Medical Center New York New York USA; ^6^ Department of Clinical Sciences Swedish University of Agricultural Sciences Uppsala Sweden; ^7^ Department of Medicine and Epidemiology, School of Veterinary Medicine University of California Davis Davis California USA; ^8^ Department of Veterinary Clinical Sciences, College of Veterinary Medicine The Ohio State University Columbus Ohio USA

**Keywords:** arrhythmogenic, cardiovascular, congestive heart failure, consensus statement, echocardiography, feline, heart, hypertrophic cardiomyopathy, restrictive cardiomyopathy, review, treatment

## Abstract

Cardiomyopathies are a heterogeneous group of myocardial disorders of mostly unknown etiology, and they occur commonly in cats. In some cats, they are well‐tolerated and are associated with normal life expectancy, but in other cats they can result in congestive heart failure, arterial thromboembolism or sudden death. Cardiomyopathy classification in cats can be challenging, and in this consensus statement we outline a classification system based on cardiac structure and function (phenotype). We also introduce a staging system for cardiomyopathy that includes subdivision of cats with subclinical cardiomyopathy into those at low risk of life‐threatening complications and those at higher risk. Based on the available literature, we offer recommendations for the approach to diagnosis and staging of cardiomyopathies, as well as for management at each stage.

**Abbreviations**
ACE: angiotensin converting enzyme
ACVIM: American College of Veterinary Internal Medicine
AHA: American Heart Association
ARVC: arrhythmogenic right ventricular cardiomyopathy
ATE: arterial thromboembolism
CHF: congestive heart failure
cTnI: cardiac troponin‐I
DCM: dilated cardiomyopathy
DLVOTO: dynamic left ventricular outflow tract obstruction
ESC: European Society of Cardiology
HCM: hypertrophic cardiomyopathy
LA: left atrial
LMWH: low‐molecular‐weight heparin
LOE: level of evidence
LV: left ventricle
NT‐proBNP: N‐terminal pro‐brain natriuretic peptide
RCM: restrictive cardiomyopathy
SAM: systolic anterior motion
SEC: spontaneous echocardiographic contrast
UCM: unclassified cardiomyopathy

## 1 INTRODUCTION

Cardiomyopathies are a heterogeneous group of myocardial diseases with variable phenotype and prognosis. Cardiomyopathies are common in cats, and cardiovascular disease is among the 10 most common causes of death in cats.^1^, ^2^, ^3^ The following report by the American College of Veterinary Internal Medicine consensus statement panel on cardiomyopathy in cats proposes an updated classification of cardiomyopathies based on echocardiographic phenotype, and provides recommendations for the diagnostic approach and management of cats with myocardial disease.

## 2 CONSENSUS METHODS

A modified Delphi method was applied to a series of statements produced by members of the committee that summarized the most important issues related to cardiomyopathy in cats. A combination of online anonymous voting with free text comments, face‐to‐face meetings and video‐conferences was used to modify the statements. Consensus was defined as ≧6 of the 9 committee members agreeing with a statement. A PubMed search using the MeSH terms “feline” and “cardiomyopathies” yielded 475 references, and further references were identified using other databases and other search terms. References documenting peer‐reviewed published studies containing original data were reviewed by the panel and graded. For each statement for which consensus was reached, a level of evidence (low/medium/high), was determined based on review of the literature (Table 1), and a class (strength) of recommendation was assigned (is recommended/should be considered/may be considered/is not recommended) according to the results of voting (Table 2).

**Table 1:** Levels of evidence

Levels of evidence	
High	Randomized controlled trials in cats
	Prospective, nonrandomized controlled trials in cats, with adequate sample size and lacking major methodological flaws
Medium	Experimental laboratory trials in cats
	Retrospective clinical studies with intervention & control groups
Low	Case series in cats without control groups
	Studies in other species
	Expert opinion

**Table 2:** Class of recommendations

Class of recommendations	
Evidence/agreement that intervention is beneficial/useful/effective (Class I)	“is recommended/indicated”
Evidence/opinion in favor of usefulness/efficacy (Class IIa)	“should be considered”
Evidence/opinion less well established (Class IIb)	“may be considered”
Evidence/agreement that intervention is not useful/effective and in some instances may be harmful (Class III)	“is not recommended”

## 3 DEFINITIONS AND CLASSIFICATION OF CARDIOMYOPATHIES IN CATS

**Figure 1:**
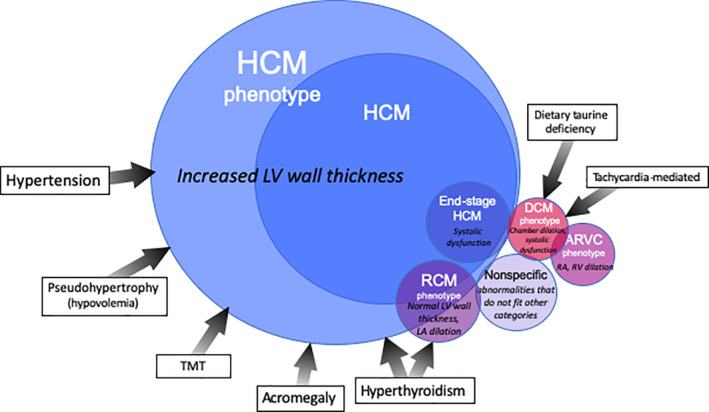
Classification of cardiomyopathy phenotypes. (adapted with permission from Clinical Small Animal Internal Medicine, Ed David Bruyette, John Wiley & Son). ARVC, arrhythmogenic right ventricular cardiomyopathy; DCM, dilated cardiomyopathy; End‐stage HCM, HCM with systolic dysfunction; HCM, hypertrophic cardiomyopathy; RCM, restrictive cardiomyopathy; TMT, transient myocardial thickening

Cardiomyopathy is defined as a myocardial disorder in which the heart muscle is structurally and functionally abnormal in the absence of any other cardiovascular disease sufficient to cause the observed myocardial abnormality.^4^ Classification of cardiomyopathies in cats previously has been based on schemes that were applied to cardiomyopathy in humans, but currently several competing classification systems are used in humans, highlighting the difficulties inherent in cardiomyopathy classification.^4^, ^5^, ^6^, ^7^ The aim of disease classification is to categorize conditions according to logical principles, such as by organ involvement, pathophysiology, phenotype, or underlying cause. An ideal classification system would standardize terminology and facilitate clinical management, but all classification systems have limitations, and this is particularly true when the underlying causes of disease are unknown. In humans, the cause of cardiomyopathy often can be determined, but this is rarely true in cats. We propose an adaptation of the European Society of Cardiology (ESC) classification^4^ for use in cats, because this scheme is based on phenotypic features without assumptions regarding underlying cause, and it focuses on a clinical, rather than genetic, approach to these disorders (Figure 1, Table 3). The ESC classification is based on the traditional phenotypic categories of hypertrophic cardiomyopathy (HCM), restrictive cardiomyopathy (RCM), dilated cardiomyopathy (DCM), unclassified cardiomyopathy (UCM), and arrhythmogenic right ventricular cardiomyopathy (ARVC), and we recommend retaining these categories (with the exception of UCM) as a basic framework, while acknowledging their limitations. For example, in some cats, the cardiac phenotype changes over time, because of disease progression, comorbidities or unknown factors.

**Table 3:** Definitions of cardiomyopathy phenotypes. Cardiomyopathy is defined as a myocardial disorder in which the heart muscle is structurally and functionally abnormal in the absence of any other disease sufficient to cause the observed myocardial abnormality

Phenotype	Definition
Hypertrophic cardiomyopathy (HCM)	Diffuse or regional increased LV wall thickness with a nondilated LV chamber.
Restrictive cardiomyopathy (RCM)	
Endomyocardial form	Characterized macroscopically by prominent endocardial scar that usually bridges the interventricular septum and LV free wall, and may cause fixed, mid‐LV obstruction and often apical LV thinning or aneurysm; LA or biatrial enlargement is generally present.
Myocardial form	Normal LV dimensions (including wall thickness) with LA or biatrial enlargement
Dilated cardiomyopathy (DCM)	LV systolic dysfunction characterized by progressive increase in ventricular dimensions, normal or reduced LV wall thickness, and atrial dilatation.
Arrhythmogenic cardiomyopathy (AC), also known as arrhythmogenic right ventricular cardiomyopathy (ARVC) or dysplasia (ARVD)	Severe RA and RV dilatation and often, RV systolic dysfunction and RV wall thinning. The left heart may also be affected. Arrhythmias and right‐sided congestive heart failure are common.
Nonspecific phenotype	A cardiomyopathic phenotype that is not adequately described by the other categories; the cardiac morphology and function should be described in detail

We propose a classification of cardiomyopathies in cats based on structural and functional characteristics, or phenotype. The phenotypic categories include cats with cardiomyopathy of both known causes (eg, hyperthyroidism, sarcomeric gene mutation) and unknown causes (most cats with a cardiomyopathy phenotype). Until an underlying cause is sought, a cat is said to have a “hypertrophic cardiomyopathy phenotype” or a “dilated cardiomyopathy phenotype” (according to the cardiac morphology and function). If no underlying cause is found, a cat is said to have “hypertrophic cardiomyopathy (HCM)” or “dilated cardiomyopathy (DCM),” as appropriate. The proposed classification therefore does not define specific disease entities, but phenotypic categories instead. The description in any individual cat can be further refined by details of cause when known. Thus, a cat with left ventricular (LV) hypertrophy and hyperthyroidism is said to have an HCM phenotype in conjunction with hyperthyroidism.

Some cats have myocardial disease that does not fit well into any category. Rather than describe these cases as having “unclassified cardiomyopathy,” according to the proposed classification these cats should be described as having cardiomyopathy with a “nonspecific phenotype.” This term always should be accompanied by a description of the morphologic and functional features to characterize the phenotype in more detail.

### 3.1 Staging cardiomyopathies in cats

**Figure 2:**
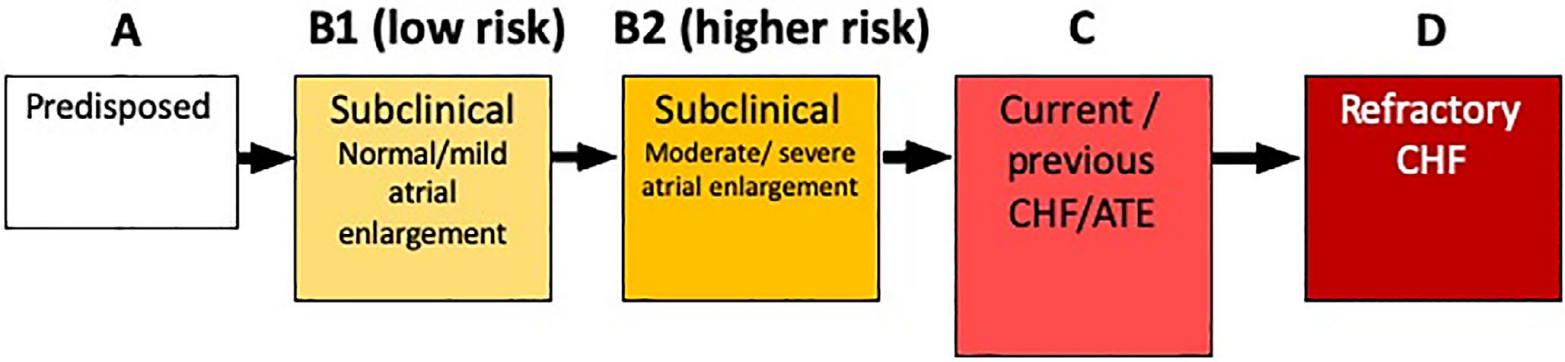
Stages of feline cardiomyopathy. Within stage B2, additional risk factors include a gallop sound, arrhythmia, decreased left atrial function, extreme left ventricular hypertrophy, left ventricular systolic dysfunction, spontaneous echo‐contrast/thrombus, regional wall motion abnormalities. ATE, arterial thromboembolism; CHF, congestive heart failure

For describing the clinical impact of cardiomyopathy in affected cats, we propose a staging system adapted from the American Heart Association (AHA) and American College of Veterinary Internal Medicine (ACVIM) heart disease staging systems^8^, ^9^, ^10^ (Figure 2), with the aim of providing a framework for prognostication and therapeutic decision‐making. Stage A includes cats that are predisposed to cardiomyopathy but have no evidence of myocardial disease. Stage B describes cats with cardiomyopathy but without clinical signs. Stage B is further divided into stage B1: cats at low risk of imminent congestive heart failure (CHF) or arterial thromboembolism (ATE), and stage B2: cats at higher risk of imminent CHF or ATE. Atrial size is an important prognostic marker, and it can be used as a means of subdividing cats with subclinical cardiomyopathy into low risk (B1) and higher risk (B2) cats. The more severe the left atrial (LA) enlargement, the higher the risk of CHF and ATE.^11^ Other factors also should be taken into consideration when staging cats with subclinical cardiomyopathy, such as LA and LV systolic function, and extreme LV hypertrophy, among others (see Figure 2). Cats that have developed signs of CHF or ATE are classified as stage C, even if clinical signs resolve with treatment. Cats with signs of CHF refractory to treatment are classified as stage D.

## 4 PREVALENCE AND NATURAL HISTORY

The most common type of cardiomyopathic phenotype is HCM and thus it is the major focus in these guidelines, but other phenotypes will be addressed separately where appropriate. Hypertrophic cardiomyopathy has an estimated prevalence of approximately 15% in the general cat population.^12^, ^13^, ^14^, ^15^, ^16^ In older cats, the prevalence is much higher, with up to 29% reported affected, even when cats with hypertension and hyperthyroidism are excluded.^15^ Most cats with HCM have subclinical disease, with a 5‐year cumulative incidence of cardiac mortality of approximately 23%, independent of age at diagnosis.^17^ Congestive heart failure (CHF) is the most common cause of clinical signs in cats with HCM, followed by arterial thromboembolism (ATE).^17^ A minority of cats die suddenly without prior clinical signs.^11^, ^17^, ^18^ The prevalence of other cardiomyopathy phenotypes in the general cat population is not known, but a hypertrophic phenotype appears to predominate in cats with subclinical cardiomyopathy.^15^

Compared with normal cats, cats with HCM are more likely to be older, male, and have a loud systolic murmur, although HCM still can be seen in young cats, females, and in cats without a murmur.^15^, ^19^, ^20^, ^21^, ^22^ Most cats with HCM are nonpedigree, but some pedigree breeds are believed to be at increased risk, including Maine Coon, Ragdoll, British Shorthair, Persian, Bengal, Sphynx, Norwegian Forest cat, and Birman breeds.^20^, ^23^, ^24^, ^25^, ^26^, ^27^ However, comprehensive prevalence data are lacking for pedigree cats. Sarcomeric gene mutations are common in people with HCM, but only 2 mutations have been identified in cats, both in the myosin binding protein C (MyBPC3) gene.^23^, ^28^ The estimated prevalence of the MyBPC3‐A31P mutation in Maine Coon cats is approximately 35% to 42%,^29^, ^30^ which is substantially higher than the prevalence of the HCM phenotype in this breed.^30^ Maine Coon cats that are homozygous for this mutation, Ragdolls homozygous for the MyBPC3‐R820W mutation, and first‐degree relatives of affected cats are at higher risk of developing HCM.^30^, ^31^, ^32^, ^33^ The role of nongenetic and epigenetic factors in HCM in cats is unknown, although such factors might be important in humans with HCM.^34^

### 4.1 Prognosis

Some cats with an HCM phenotype remain subclinical, whereas others develop CHF or ATE.^13^, ^17^, ^19^, ^20^, ^21^, ^32^, ^35^, ^36^, ^37^ Younger age^22^ and lack of clinical signs^19^, ^20^, ^22^, ^35^ have been associated with longer survival. Markers of increased risk of CHF or ATE include presence of a gallop sound or arrhythmia on physical examination, moderate to severe LA enlargement, decreased LA fractional shortening (LA FS%), extreme LV hypertrophy, decreased LV systolic function, spontaneous echo‐contrast or intracardiac thrombus, regional wall thinning with hypokinesis, and a restrictive diastolic filling pattern.^11^, ^22^, ^38^ Sudden cardiac death also occurs in cats with HCM.^17^, ^18^, ^21^ Less is known about risk factors for sudden cardiac death, but these might include a history of syncope, ventricular arrhythmias, LA enlargement, and regional LV wall hypokinesis.^11^ Median survival times are substantially shorter in cats with HCM that develop CHF or ATE compared to those with subclinical cardiomyopathy.^17^, ^19^, ^20^, ^21^, ^22^, ^35^ Cats that develop CHF associated with stress, IV fluid therapy, general anesthesia, or extended‐release corticosteroid treatment might have longer survival times compared with cats that develop CHF in the absence of these factors.^3^, ^17^, ^39^ Factors associated with longer survival times after treatment for CHF include a greater decrease in NT‐proBNP concentrations during hospitalization and resolution of CHF at reexamination.^40^

In contrast to HCM in people, in whom dynamic left ventricular outflow tract obstruction (DLVOTO) has been associated with increased morbidity and mortality,^41^ DLVOTO does not appear to be a poor prognostic factor in cats.^17^, ^21^, ^22^ This might be a true difference between HCM in humans and cats, but also could reflect differences in how DLVOTO is defined between species, or the result of bias in retrospective studies (cats with DLVOTO are more likely to be investigated for an incidentally detected murmur than cats with nonobstructive HCM, which often are not diagnosed until clinical signs develop).

## 5 DIAGNOSIS

Establishing the diagnosis of cardiomyopathy in cats can be challenging, particularly in general practice. Echocardiography performed by a veterinary cardiology specialist is the diagnostic test of choice, but differentiation of the various phenotypic categories sometimes can be challenging, even for specialists. Fortunately, with regard to therapeutic decision‐making, the requirement for diuretics in cats with CHF and the approach to management of ATE are similar regardless of the form of cardiomyopathy. A basic level of echocardiographic skill (eg, ability to detect moderate to severe left atrial enlargement) can be sufficient to identify the more advanced stages of cardiomyopathy.^42^, ^43^ Other diagnostic tests may facilitate disease staging, identification of important comorbidities and establishing prognosis. Importantly, tests are recommended to screen for a possible underlying cause for the cardiomyopathy phenotype, such as serum thyroxine concentration for hyperthyroidism or blood pressure measurement for systemic hypertension in cats with an HCM phenotype.

### 5.1 Genetic testing

Genetic testing for the MyBPC3‐A31P mutation and the MyBPC3 R820W mutation is recommended in Maine Coon and Ragdoll cats (respectively) intended for breeding (level of evidence [LOE] high), with the aim of decreasing the incidence of these mutations and HCM in these breeds.^29^, ^30^, ^44^ It is recommended that cats homozygous for either mutation not be used for breeding, but heterozygous cats can be bred to genotype‐negative cats if they have other outstanding characteristics (LOE low). The same genetic tests can be considered in nonbreeding Maine Coon or Ragdoll cats to determine the relative risk of developing HCM (LOE medium). In Maine Coon cats it is primarily individuals that are homozygous for the A31P mutation that develop clinically relevant HCM.^44^ Maine Coon and Ragdoll cats that test negative for these MYBPC3 mutations have been reported with HCM (LOE high),^44^, ^45^ and thus regular echocardiographic screening should be considered even in Maine Coon and Ragdoll cats without these mutations (LOE low). Genetic testing for the A31P and R820W MYBPC3 mutations in non‐Maine Coon or non‐Ragdoll cats is not recommended, because these 2 mutations are almost completely specific to Maine Coon and Ragdoll cats (LOE high).^29^, ^30^

### 5.2 History

The history is unremarkable in many cats with cardiomyopathy, particularly those with HCM. The most common presenting sign is labored breathing,^17^ although some cats only show nonspecific signs such as hiding or inappetence. Congestive heart failure appears to be the most common cause of respiratory distress in cats.^46^, ^47^ Paresis or paralysis associated with ATE is also a common presenting sign,^17^ with syncope being less common.^19^ In some cats with cardiomyopathy, sudden death may occur with no premonitory signs.^18^

### 5.3 Physical examination

#### 5.3.1 Subclinical cardiomyopathy

A parasternal systolic heart murmur has been reported in up to 80% of cats with subclinical HCM, compared with 30%‐45% of healthy cats without HCM.^14^, ^15^, ^16^, ^17^ Third heart sounds such as gallop sounds have been reported in 2.6%‐19% of cats with subclinical HCM and are seldom present in healthy cats.^15^ Arrhythmias also can be associated with cardiomyopathies.^48^, ^49^ Many affected cats have no auscultatory abnormality.^15^, ^42^ Further investigations are recommended if a heart murmur is detected in any cat (LOE medium).^15^, ^42^ A loud systolic murmur (grade 3‐4/6) is more common in cats with HCM than in normal cats, but an increase in heart murmur intensity over time does not necessarily indicate the presence or worsening of disease. A palpable thrill (grade 5‐6/6 murmur) seldom is associated with cardiomyopathy in cats and is more likely to be associated with a congenital malformation. Cats with more advanced disease (or those with restrictive or dilated phenotypes) may not have an audible murmur.^13^, ^21^, ^42^, ^50^ Auscultation of a gallop sound or an arrhythmia is more likely to be associated with cardiomyopathy, although differentiation of a gallop sound from other third heart sounds or a bigeminal rhythm can be challenging.

#### 5.3.2 Cardiomyopathy associated with CHF

Tachypnea, labored breathing or both are the typical historical and physical examination findings in cats with left heart failure. Compared with cats that have subclinical HCM, a gallop sound or an audible arrhythmia are more common in cats with CHF, and murmurs are less common.^13^, ^21^, ^43^ In one study, cats presented to first opinion practices for evaluation of respiratory distress with respiratory rates >80 breaths/min, gallop sounds, rectal temperatures <37.5°C or heart rates >200 bpm were more likely to have CHF than other causes of dyspnea.^47^ Pulmonary crackles can be heard when pulmonary edema is present, and breath sounds are often diminished ventrally when pleural effusion is present, together with paradoxical breathing.^51^

### 5.4 Radiography

Cardiomyopathy might be suspected when severe cardiomegaly is present radiographically, when a left auricular bulge is present on dorsoventral/ventrodorsal radiographic views, or both.^52^, ^53^ Thoracic radiography is insensitive for identification of mild or moderate cardiac changes associated with cardiomyopathy, and in some cats the cardiac silhouette may appear normal even when disease is severe enough to cause CHF.^54^ Furthermore, it is difficult to identify the cardiomyopathy phenotype from the shape of the cardiac silhouette, and the classic “valentine‐shaped” heart is not specific for HCM, as previously thought.^55^, ^56^ Although radiography is considered the gold standard for confirming the presence of cardiogenic pulmonary edema, if radiographs cannot be obtained safely consideration should be given to delaying thoracic radiography (LOE low). In contrast to dogs, the radiographic pattern associated with cardiogenic pulmonary edema in cats is highly variable.^52^, ^57^ A combination of physical examination, point‐of‐care ultrasound examination and point‐of‐care NT‐proBNP often can be helpful when deciding if CHF is the cause of respiratory distress (LOE high).^58^

### 5.5 Cardiac biomarkers

#### 5.5.1 NT‐proBNP

The quantitative feline‐specific NT‐proBNP assay using plasma or pleural fluid has good diagnostic accuracy for discriminating between cats with cardiac and noncardiac causes of respiratory distress (LOE high),^59^, ^60^, ^61^, ^62^, ^63^, ^64^ but it is not recommended for guiding therapeutic decision‐making in cats with respiratory distress because of the delay in receiving test results from an external laboratory. Instead, a point‐of‐care NT‐proBNP assay provides rapid results while maintaining reasonable diagnostic accuracy in discriminating between cardiac and noncardiac causes of respiratory distress, and should be considered when point‐of‐care ultrasound examination is not available (LOE medium).^58^, ^65^ The point‐of‐care assay can be used on plasma or pleural fluid,^64^, ^66^ the latter diluted 1:1 with saline for greater specificity.^65^

When investigating a cat suspected to have subclinical cardiomyopathy there is less urgency for test results, and so the quantitative NT‐proBNP assay can be considered in situations where echocardiography is not available (LOE high). The quantitative NT‐proBNP assay is not recommended for differentiating normal cats from cats with mild to moderate HCM (LOE high).^67^, ^68^, ^69^ The point‐of‐care assay can be considered in cats with suspected subclinical cardiomyopathy, but its principal value is in differentiating cats with severe subclinical cardiomyopathy from normal cats or cats with only mild disease (LOE high).^70^, ^71^

#### 5.5.2 Troponin‐I

Measuring circulating cardiac troponin‐I (cTnI) concentrations can help discriminate between cardiac and noncardiac causes of respiratory distress (LOE medium), but only when results can be obtained rapidly.^72^, ^73^, ^74^ High‐sensitivity assays for human cTnI might be considered for differentiating between normal cats and cats with subclinical HCM when cardiac disease is suspected (LOE medium).^75^, ^76^ In addition, cTnI also might be considered for its prognostic value, because an increased circulating cTnI concentration is associated with increased risk of cardiovascular death independent of LA size (LOE high).^77^

### 5.6 Electrocardiography

The sensitivity of a 6‐lead ECG for detecting LV hypertrophy or LA enlargement is low,^13^, ^78^, ^79^ and ECG is not recommended as a screening method for cardiomyopathies in cats (LOE medium), despite its use in screening people for HCM.^80^ Nevertheless, a variety of arrhythmias can occur in cats with cardiomyopathy,^48^, ^49^, ^78^, ^81^, ^82^, ^83^, ^84^, ^85^ and can contribute to clinical signs such as weakness, syncope, and hypoxic‐anoxic seizures.^86^, ^87^ Although ambulatory (Holter) ECG monitoring is tolerated less well by cats than dogs, it can identify arrhythmias that might otherwise go undetected.^49^, ^88^ It is recommended that cats experiencing episodic weakness and collapse (including seizure‐like activity) should undergo a cardiovascular evaluation that includes echocardiography, ECG, and telemetric or Holter ECG monitoring if necessary. Implantable loop recorders also should be considered for cats with intermittent clinical signs that could be attributed to arrhythmias^89^, ^90^ (LOE low). Other options for recording cardiac rhythm in the cat's home environment include use of a portable electrode plate (Kardia AliveCor) in conjunction with a smartphone to record an ECG that can be interpreted by a specialist.

### 5.7 Blood pressure

Diffuse or segmental LV hypertrophy is common in cats with systemic hypertension and is observed in up to 85% of cases, although HCM and systemic hypertension may exist concurrently. For many hypertensive cats, LV hypertrophy is only mild to moderate.^91^, ^92^, ^93^, ^94^ Blood pressure determination should be considered for all cats with increased LV wall thickness (LOE medium).

### 5.8 Thyroxine measurement

Hyperthyroidism is common in older cats, and is associated with auscultatory abnormalities (murmur, gallop, arrhythmias), cardiac remodeling (LV hypertrophy or increased cardiac chamber diameters), and in some cases, CHF or ATE.^95^, ^96^, ^97^, ^98^ Hyperthyroid cats with severe LV hypertrophy are sometimes seen, but this association is suspected to be the result of hyperthyroidism exacerbating preexisting mild to moderate HCM (LOE low). It is recommended that serum thyroxine concentrations be measured in all cats ≧6 years of age with abnormal cardiac auscultation findings with or without LV hypertrophy on an echocardiogram (LOE low).

### 5.9 Echocardiography

Echocardiography is the gold standard test for diagnosis of cardiomyopathies in cats. Indications for echocardiography are listed in Table 4. Echocardiography ideally should be performed by trained operators^99^ in unsedated cats in quiet conditions, and cats should be handled with minimal restraint. When necessary, sedation of the cat may be considered for echocardiographic examination.^100^ Adequate echocardiographic images can be obtained whether the cat is in lateral recumbency or standing.^99^

**Table 4:** Main indications for cardiac evaluation

History	Syncope Seizures (in the absence of other neurological abnormalities) Diagnosis of cardiomyopathy in a close relative Weakness Exercise intolerance/open‐mouth breathing with exertion Intolerance to parenteral fluid administration Pedigree cat intended for breeding Maine coon or Ragdoll with a MyBPC3 mutation Any endocrinopathy Heartworm positive status Fever of unknown origin
Physical exam	Murmur Gallop sound or systolic click Muffled heart or lung sounds Arrhythmia Tachypnea Pulmonary crackles Jugular venous distention or pulsation Ascites Hypo‐ or hyperkinetic femoral arterial pulse pressure Acute paresis/paralysis Absent femoral arterial pulses
Cats aged 9 years or older undergoing interventions that could precipitate CHF	General anesthesia Fluid treatment Extended‐release glucocorticosteroids

Measurements of LV wall thickness traditionally have been made from 2D‐guided M‐mode echocardiographic images, although this mode is limited to focal sampling of the LV. Because of regional heterogeneity of LV hypertrophy in many cats with HCM, measurements using M‐mode echocardiography can miss focal wall thickening. Inadvertent measurement of papillary muscles also is possible as a result of translational motion of the heart. Two‐dimensional echocardiography allows measurement of LV wall thickness in multiple locations. The frame rate should be sufficiently high (>40 Hz) to allow measurement of the true end‐diastolic wall thickness. Measurements of LV wall thickness currently are made using 2D, M‐mode or both, but the 2 techniques can yield different values for wall thickness and are not interchangeable.^16^

If M‐mode echocardiography is used, it is recommended that it be guided by a 2D short‐axis view (LOE low). With 2D echocardiography, it is recommended that end‐diastolic LV wall thicknesses be measured in at least 2 right parasternal views (long axis and short axis), measuring the thickest part of the septum and free wall in each view (LOE low). Using 2D‐guided M‐mode, it is recommended that septal and free wall thicknesses be measured using a leading edge‐to‐leading edge technique, so that for the septum, the LV endocardial layer is excluded, and for the free wall, the pericardium is excluded (LOE low).^101^ Using 2D echocardiography, a leading edge‐to‐trailing edge technique should be considered for measuring the septum, and a leading edge‐to‐leading edge technique (excluding the pericardium) for measuring the LV free wall (LOE low). In regions in which marked endocardial thickening occurs, it is recommended that the endocardial layer be excluded from the measurements (LOE low). It is recommended that end‐diastolic LV wall thickness be measured from at least 3 cardiac cycles and averaged (LOE low). End‐diastolic and end‐systolic LV diameters traditionally have been measured using M‐mode (using a leading edge‐to‐leading edge technique), but also can be measured from 2D images (using a trailing‐to‐leading edge technique). Left ventricular fractional shortening is the most commonly used quantitative index of LV systolic function, and regional wall motion abnormalities usually are noted as a subjective finding. Papillary muscle size and geometry are evaluated from right parasternal long and short axis images and usually are described qualitatively, although they can be quantitatively assessed.^102^

Left atrial size can be measured in short axis and long axis views. Left atrial diameter can be measured in a right parasternal short‐axis view that includes the aortic valve cusps, and can be indexed to aortic diameter (LA/Ao) in the same frame. Measurements can be made either at end‐systole^103^ or end‐diastole.^104^ Reference intervals will vary according to the timing within the cardiac cycle (LOE medium). The LA diameter also can be measured in a right parasternal long‐axis 4‐chamber view at end‐systole, from the interatrial septum to the LA free wall (LOE medium).^105^ Left atrial fractional shortening is an index of LA function, and can be measured using 2D‐guided M‐mode from the same right parasternal short axis view as used for measurement of LA/Ao.^103^ The presence of spontaneous echo‐contrast (SEC) is associated with decreased LA function and blood stasis, and it is recommended that the LA appendage be evaluated in cats with LA enlargement for the presence of SEC or thrombus^106^ and for evidence of blood stasis using pulsed wave Doppler LA appendage flow velocities (LOE Low).

In addition to measuring cardiac chamber dimensions, it is recommended that the presence or absence of dynamic left ventricular outflow tract obstruction (DLVOTO) be evaluated. Assessment of DLVOTO can be made using a combination of 2D, M‐mode, and Doppler echocardiography. Careful imaging of the LVOT using 2D echocardiography should allow identification of systolic anterior motion of the mitral valve (SAM), where the septal leaflet of the mitral valve is displaced towards the septum in systole, obstructing the LVOT. Chordal anterior motion (CAM) also can be identified in the same imaging view.^107^ Mitral valve SAM also can be imaged using M‐mode echocardiography. Color Doppler can be used to identify the characteristic blood flow jets of LVOT obstruction and mitral regurgitation, and spectral Doppler can be used to estimate the peak LVOT gradient from left apical views (LOE Low). It is recommended that diastolic function be assessed and a class of diastolic dysfunction assigned using a combination of spectral Doppler and tissue Doppler imaging (LOE medium).^108^, ^109^ Table 5 summarizes recommended echocardiographic protocols.

**Table 5:** Echocardiographic protocols for a cat suspected of having cardiomyopathy according to level (basic to advanced)

Level of scan	Measurements	Qualitative assessment
Focused point‐of‐care		Note presence of: Pleural, pericardial effusions Left atrial size & motion Pulmonary B‐lines LV systolic function
Standard of care	*M‐mode* IVSd, LVFWd LVIDd, LVIDs, LV FS% LA FS% *2D* IVSd, LVFWd LVIDd, LVIDs LA/Ao LA diameter from RP long axis view	Note presence of: Papillary muscle hypertrophy End‐systolic LV cavity obliteration Papillary muscle/mitral leaflet abnormalities SAM or mid LV obstruction Dynamic RVOTO Abnormal cardiac chamber geometry Presence of spontaneous echo‐contrast or thrombus Regional wall motion abnormalities
Best practice	M‐mode and 2D as for standard of care, with the following additional measurements: *Spectral Doppler* Mitral inflow velocities Isovolumic relaxation time LVOT velocities RVOT velocities PVF velocities LAA blood flow velocities *Tissue Doppler imaging* Lateral and septal mitral annular velocities (pulsed wave Doppler mode).	Qualitative assessment as for standard of care

*Note:* “Focused point‐of‐care” scan: an abbreviated echocardiographic examination conducted because of patient instability, because the operator has limited training in echocardiography, or both; “standard of care” scan: an echocardiographic examination that includes the content considered to be standard by a trained, competent observer; “best practice” scan: an echocardiographic examination conducted by a cardiologist with particular expertise in echocardiography. IVSd: end‐diastolic interventricular septal thickness, LA: left atrial, LA FS%: left atrial fractional shortening, LA/Ao: left atrial to aortic ratio at end‐diastole and end‐systole, or both, LAA: left atrial appendage, LV: left ventricular, LV FS%: left ventricular fractional shortening, LVFWd: end‐diastolic left ventricular free wall thickness, LVIDd: left ventricular internal dimension at end‐diastole, LVIDs: left ventricular internal dimension at end‐systole, LVOT: left ventricular outflow tract, PVF: pulmonary venous flow, RP: right parasternal, RVOT: right ventricular outflow tract, SAM: systolic anterior motion of the mitral valve.

#### 5.9.1 Echocardiographic protocol for cardiomyopathy screening in pedigree breeding cats

A standard‐of‐care scan should be undertaken at a minimum for screening pedigree breeding cats. Such a scan consists of a quantitative assessment of left heart chamber dimensions, including LA size, LV wall thickness and LV diameter, as well as LA and LV fractional shortening and a qualitative assessment of abnormal cardiac chamber geometry and presence or absence of SAM of the mitral valve (Table 5). No reference interval for maximal end‐diastolic LV wall thickness is universally accepted, and it is overly simplistic to expect a single cutoff value for wall thickness to differentiate a normal ventricle from a hypertrophied ventricle. Furthermore, wall thickness increases with increasing body size,^26^, ^110^, ^111^ and is influenced by hydration^112^, ^113^ and heart rate.^114^ For the majority of normally‐sized cats, an end‐diastolic LV wall thickness <5 mm is considered normal, and ≧6 mm is indicative of hypertrophy. It is recommended that LV wall thicknesses between 5 and 6 mm should be interpreted in the context of body size, family history, qualitative assessment of LA and LV morphology and function, presence of DLVOTO and tissue Doppler imaging velocities. Where there is doubt, it is recommended that the cat be classified as equivocal for LV hypertrophy and reevaluated at a later date.

#### 5.9.2 Echocardiographic protocol for a cat suspected to have cardiomyopathy

Further investigations are recommended when history, physical examination findings, or both suggest that a cat might have cardiomyopathy (Table 4, LOE medium). Further investigations also should be considered in older cats when anesthesia or treatment with IV fluid therapy or extended‐release corticosteroids is planned (LOE low). It is recommended that a standard of care examination include a qualitative evaluation of SEC and regional wall motion abnormalities (Table 5). A best practice examination includes the above evaluations and Doppler blood flow velocities recorded in the LVOT, across the mitral valve, in the pulmonary veins, and in the LA appendage. Mitral annulus velocities also should be recorded with tissue Doppler imaging. If a standard‐of‐care assessment is not possible, a focused point‐of‐care examination still can provide some information on the presence of disease and risk of CHF or ATE based on a qualitative assessment of LA size and cardiac chamber geometry.

#### 5.9.3 Echocardiographic protocol for a cat suspected to have congestive heart failure

For clinically unstable cats or where specialist level echocardiography is not available, a focused point‐of‐care examination can be used to identify the presence of pleural or pericardial fluid or both, presence of B lines in lungs, and to provide a subjective estimate of LA size and LV systolic function (Table 5).^58^ It is recommended that this examination be followed by a best practice examination or at least a standard‐of‐care examination once the cat is more stable, using the protocol suggested for cats with suspected cardiomyopathy.

### 5.10 Approach to the diagnosis of subclinical cardiomyopathy

Cats with subclinical cardiomyopathy can be difficult to identify. Cardiac evaluation should be considered for cats with a suspicious history or physical examination findings that include a gallop sound, murmur, or arrhythmia, and in cats judged to be at high risk of CHF if subjected to interventions such as anesthesia or IV fluid therapy (LOE low; Table 4). Echocardiography is currently the most accurate clinical test for diagnosis of cardiomyopathy in cats, and is also the best technique for estimating prognosis, but is highly user‐dependent.^99^ However, with appropriate training and experience, focused point‐of‐care echocardiography is feasible in first opinion (general) practice and can be used to improve the accuracy of cardiomyopathy diagnosis by nonspecialist practitioners, particularly in cats with more advanced disease.^42^ It is recommended that focused point‐of‐care echocardiography be undertaken only after appropriate training and practice^42^, ^99^ (LOE high) and a point‐of‐care examination should be followed at a later time point with a standard echocardiographic examination to characterize the phenotype.

When echocardiography is unavailable, evaluation of NT‐proBNP concentrations may be considered. Circulating NT‐proBNP concentrations increase with increasing clinical severity of cardiomyopathy in groups of cats, but overlap precludes using NT‐proBNP to categorize individual cats into mild, moderate, and severe groups.^69^ The measurement of NT‐proBNP can be considered as an initial screening test for identifying advanced cardiomyopathy. Normal NT‐pro‐BNP results do not assure that a cat is free of cardiomyopathy, especially when mild heart disease is present, nor do they guarantee that a cat will remain free of cardiomyopathy later in life. They do however indicate a low likelihood of cardiomyopathy that is immediately, clinically harmful. Therefore, in a cat suspected of cardiomyopathy, follow‐up echocardiography still should be considered, even if initial NT‐proBNP results are within normal reference intervals (LOE low). It is recommended that a positive NT‐proBNP test always be followed by an echocardiographic examination.

In older cats with heart murmurs, gallop sounds or arrhythmias, it is recommended that serum T4 concentration and blood pressure be measured (LOE high). Echocardiography also should be considered (LOE low).

### 5.11 Approach to diagnosis in cats with suspected CHF

Physical examination findings of tachypnea, labored breathing, respiratory crackles, hypothermia, and a gallop sound are highly suggestive of CHF,^47^ but in some cats tachypnea with labored breathing might be the only abnormality detected. Although thoracic radiography traditionally has been considered the gold standard test for detecting cardiogenic pulmonary edema, care should be taken to minimize stress when taking radiographs of cats with respiratory distress. Pulmonary infiltrates and cardiomegaly are the key findings with CHF, but classic radiographic features of CHF such as LA enlargement and distended pulmonary vessels are inconsistently identified in affected cats.^52^, ^54^

If radiographs cannot be obtained safely, point‐of‐care thoracic ultrasound examination or a point‐of‐care NT‐proBNP test should be considered (LOE high). With point‐of‐care ultrasound examination, the presence of effusions or B‐lines in association with severe LA enlargement is highly suggestive of CHF.^58^, ^115^ A negative result on a point‐of‐care NT‐proBNP test suggests that respiratory disease is more likely to be the cause of respiratory distress than is cardiac disease. Once a cat with CHF has been stabilized, a standard‐of‐care or best practice echocardiographic examination should be considered (Table 5, LOE low).

## 6 TREATMENT

### 6.1 Stage B1 cardiomyopathy

Treatment of cats with subclinical cardiomyopathy is controversial because evidence is lacking. Although the majority of cats with stage B1 cardiomyopathy will not develop clinical signs, it is recommended that stage B1 cats be monitored annually for development of moderate to severe LA enlargement (progression to stage B2). Cats with stage B1 cardiomyopathy are considered at low risk of CHF or ATE, and in general treatment is not recommended (LOE low).

There is no evidence that DLVOTO is associated with increased morbidity or mortality in cats, and atenolol has not been shown to have any effect on the 5‐year survival rate in cats with subclinical HCM.^116^ However, atenolol is expected to decrease DLVOTO gradient and heart rate,^117^ and may be considered in cats with stage B1 cardiomyopathy and severe DLVOTO, provided it can be administered consistently (LOE low).

### 6.2 Stage B2 cardiomyopathy

Cats with stage B2 HCM have an increased risk of developing CHF or ATE. Primary prevention of ATE in cats with subclinical cardiomyopathy has not been studied, but thromboprophylaxis is recommended when known risk factors for ATE are present.^11^, ^106^ Clopidogrel was more effective than aspirin in cats that had survived a previous ATE episode,^118^ and no other randomized, controlled studies have been reported. Clopidogrel therefore is recommended in cats considered at risk of ATE (moderate to severe LA enlargement, low LA FS%, low LA appendage velocities, SEC; LOE medium). Clopidogrel does not eliminate the risk of ATE, and thus other antithrombotic drugs can be considered in addition to clopidogrel in cats believed to be at very high risk of ATE (eg, clopidogrel plus aspirin, clopidogrel plus a PO factor Xa inhibitor, or clopidogrel plus aspirin plus a PO factor Xa inhibitor; LOE low).

Cats with stage B2 cardiomyopathy should be monitored for progression of disease and development of clinical signs, but the effects of stress caused by reexamination also should be taken into consideration. If a stage B2 cat is reexamined, attention to appropriate handling and minimizing stressful stimuli is important. If these measures are (or are likely to be) insufficient, PO administration of appropriate pharmaceuticals,^119^, ^120^ synthetic feline pheromone application^121^, ^122^, ^123^ or both can be considered (LOE medium). Once the LA is moderately to severely enlarged and antithrombotic treatment is started, management is unlikely to change until clinical signs develop, but at a minimum, it is recommended that owners monitor the cat's resting or sleeping respiratory rate^124^ (LOE medium).

In 2 randomized, placebo‐controlled studies, neither an angiotensin converting enzyme (ACE) inhibitor (ramipril) nor spironolactone had any effect on LV mass or diastolic function in cats with subclinical HCM, but study populations were small and limited to cats of a single breed that had heritable cardiomyopathy.^125^, ^126^ Similarly, benazepril had no effect on time to treatment failure compared to placebo in a randomized placebo‐controlled study that included cats with subclinical heart disease.^127^ No studies have been reported of pimobendan use in cats with subclinical cardiomyopathy.

Ventricular ectopy is common in cats with HCM^48^, ^49^and ARVC^48^, ^128^, ^129^ and is associated with sudden death in people with these cardiomyopathies.^80^, ^129^, ^130^ Treatment options in cats are limited, but atenolol has been shown to decrease ventricular ectopy in cats with HCM.^117^ It is recommended that cats with complex ventricular ectopy be treated with atenolol (6.25 mg/cat q12h PO) or sotalol (10‐20 mg/cat q12h PO; LOE low). Markedly increased heart rate is observed in a minority of cats with atrial fibrillation (AF),^83^ but AF occurs most often in the setting of advanced cardiomyopathy where tachycardia is poorly tolerated. Diltiazem, atenolol or sotalol may be considered in cats with AF and a rapid ventricular response rate (LOE Low).

### 6.3 Stage C

#### 6.3.1 Acute decompensated heart failure

Cats with pulmonary edema or pleural effusion caused by CHF usually are presented with tachypnea and labored breathing. Empirical diuretic treatment should be considered immediately when the index of suspicion for CHF is high, for example, if hypothermia and a gallop sound are present, especially when echocardiography or thoracic radiography is unavailable or the risk of restraint for diagnostic evaluation appears to exceed the benefits (LOE low). Supplementary oxygen administration is recommended for any cat with respiratory distress, and sedation with an anxiolytic (eg, butorphanol) also should be considered (LOE low). Stress should be further minimized by gentle handling, a quiet environment, and provision of a hiding box.^131^

Intravenous administration of furosemide, either as multiple boluses of 1 to 2 mg/kg or a constant rate infusion, is recommended for CHF and pulmonary edema in particular (LOE low). Thoracocentesis should be performed when respiratory distress results from pleural effusion (LOE low). Intravenous fluid treatment is contraindicated in cats with clinically evident congestion, edema or effusion, and can exacerbate signs of CHF even if diuretics are administered concurrently (LOE low). Ideally, measuring blood chemistries can be considered before treatment if samples can be obtained without compromising patient safety (LOE low), but diuretic treatment is recommended for acute heart failure regardless of the presence of azotemia (LOE low).

In cats with signs of low cardiac output (eg, hypotension, hypothermia, bradycardia), PO treatment with pimobendan could be considered, provided DLVOTO is absent (LOE low). In cats with acute heart failure and low cardiac output signs that do not show clinical improvement after administration of pimobendan, a constant rate infusion of dobutamine could be considered (LOE low). Evidence of the efficacy of transdermal administration of nitroglycerin in cats is lacking or conflicting, and its use is not recommended (LOE medium). Angiotensin converting enzyme inhibition is not indicated during acute decompensation of cats with cardiomyopathy (LOE low). Monitoring body temperature, respiratory rate, body weight, blood pressure, and estimated urine output is recommended (LOE high).

Once stabilized, it is recommended that cats be discharged to the care of their owners as soon as possible (LOE low). Reevaluation is recommended 3‐7 days after discharge to evaluate for resolution of CHF and to evaluate renal function and serum electrolyte concentrations (LOE low). It is recommended that owners monitor the cat's resting or sleeping respiratory rate with the goal of maintaining the respiratory rate <30 breaths/min (LOE medium).

#### 6.3.2 Chronic heart failure

Furosemide is the primary drug used for control of pulmonary edema and effusions in cats with CHF. Typically, treatment consists of furosemide 0.5 to 2 mg/kg PO q8‐12 h, depending on the severity of clinical signs of CHF. A common starting dosage is 1 to 2 mg/kg PO q12h (LOE low). Intravenous administration is preferred in cats with marked respiratory distress from pulmonary edema (see treatment of acute decompensated heart failure, above). The maintenance dose of furosemide should be titrated to maintain a resting or sleeping respiratory rate at home of <30 breaths/min (LOE low). Measurement of serum creatinine, blood urea nitrogen, and electrolyte concentrations is recommended 3‐7 days after initiating furosemide (LOE low). Angiotensin converting enzyme inhibition with benazepril did not delay the onset of treatment failure in a randomized, placebo‐controlled study of cats with CHF^127^ (LOE high) although ACE inhibitors still are used by some cardiologists. Prophylactic antithrombotic treatment with clopidogrel (18.75 mg/cat PO q24h, with food) is recommended in any cat with a history of CHF and moderate to severe LA enlargement (LOE low). Some cats react to clopidogrel with salivation and retching or vomiting, which can be minimized by administering the medication in an empty gelatin capsule, followed by water. Pimobendan can be considered in cats without clinically relevant LVOTO (LOE low).^132^ A commonly used dosage is 0.625 to 1.25 mg per cat q12h PO.

It is recommended that cats with stage C cardiomyopathy be reexamined at approximately 2‐4 month intervals or as needed. Consideration should be given to the effects of stress caused by reexamination on an individual basis. Owner‐recorded resting or sleeping respiratory rate can provide useful information for adjusting medication over the phone without the need for clinic visits, although the presence of comorbidities and the risk of disease progression may necessitate periodic reexaminations. For cats with a DCM phenotype, enquiries about dietary history and measurement of plasma taurine concentrations are recommended, with supplementation and dietary change as necessary.

### 6.4 Stage D (refractory)

Torsemide may be considered in place of furosemide in cats with persistent CHF despite high doses of furosemide (>6 mg/kg/day PO), at a starting dose of 0.1 to 0.2 mg/kg PO q24h and uptitrating to effect (LOE low). Spironolactone 1 to 2 mg/kg PO q12h to q24h also can be considered for management of chronic CHF.^133^ Adverse reactions (eg, ulcerative dermatitis) have been reported in Maine Coon cats treated with spironolactone at a dosage of 2 mg/kg q12h (LOE medium).^126^ In cats with global LV systolic dysfunction, pimobendan is recommended (LOE low).^134^ Taurine supplementation at 250 mg PO q12h also is recommended for cats with global LV systolic dysfunction unless plasma taurine concentrations are in the normal range (LOE low).^135^ Foods high in salt should be avoided (LOE low). As the number of medications increases, owner compliance is likely to decrease, and unnecessary medications should be avoided (LOE low).

Cardiac cachexia, defined as loss of muscle or lean body mass associated with heart failure, may be present in cats with stage D cardiomyopathy. Calorie intake should be prioritized over restriction of sodium intake and body condition score should be recorded and an accurate body weight obtained at every clinic visit (LOE low). It is recommended that serum potassium concentration be monitored and if hypokalemia is identified, the diet should be supplemented with potassium from either natural or commercial sources (LOE low).

### 6.5 Management of ATE

Most cats with ATE presented to first opinion practice are euthanized.^136^ This approach is justifiable In terms of the cat's welfare and generally poor prognosis, but if analgesia is adequate and favorable prognostic factors are present (eg, normothermia, only 1 limb affected, absence of CHF),^136^, ^137^ an attempt at treatment can be considered provided the owner is fully informed of the risks and overall poor prognosis.

Analgesia is a priority for management of acute ATE in the first 24 hours, and treatment with a mu opioid agonist such as fentanyl, hydromorphone, or methadone is recommended (LOE low). Anticoagulant treatment is recommended using low‐molecular‐weight heparin (LMWH) or unfractionated heparin, or a PO factor Xa inhibitor, which should be started as soon as possible (LOE low). Thrombolytic treatment is not recommended for cats with ATE (LOE high).^138^, ^139^, ^140^ If CHF is present with ATE, management with furosemide and oxygen is recommended as necessary (LOE high), but it is important to note that pain also can cause tachypnea, and this should not be mistaken for the presence of CHF. It is recommended that clopidogrel be started as soon as the cat can tolerate PO medications, with an initial loading dose of 75 mg PO (LOE low) followed by 18.75 mg PO q24h (LOE high). Heparin can be replaced by a PO factor Xa inhibitor in combination with clopidogrel (LOE low).

#### 6.5.1 Post‐ATE

Reexamination is recommended 3‐7 days after discharge from the hospital, as well as 1‐2 weeks after the ATE event. Evaluation should include assessment of the distal limbs for evidence of necrosis, electrolyte status, appetite, and treatment compliance, as well as the degree of improvement in neuromuscular function. Resolution of lower motor neuron dysfunction can take weeks or months in some cats,^141^ and reexamination should be considered every 1‐3 months, considering the effects of stress in the individual cat. It is recommended that the owner continue to monitor resting or sleeping respiratory rate.

## 7 CONCLUSIONS

Cardiomyopathies in cats are a heterogeneous group of myocardial disorders of mostly unknown etiology and with potentially life‐threatening consequences. However, it is possible to identify cats at high risk of adverse events. In this consensus statement, we have outlined an approach to diagnosis and treatment that should be accessible to general practitioners as well as specialists. We make several new recommendations: cardiomyopathy classification should be focused on phenotype, but staging is more important for management than type of cardiomyopathy. Echocardiography is a very powerful tool that can provide valuable information, but even a simple focused point‐of‐care ultrasound examination can be performed by nonspecialist practitioners to identify cats at high risk of CHF or ATE, or those already presenting with CHF. Evidence‐based recommendations are provided on diagnosis and treatment of cardiomyopathies according to stage.

## CONFLICT OF INTEREST DECLARATION

Luis Fuentes: Boehringer Ingelheim Vetmedica (consultancy, speaking); CEVA (program support), IDEXX (research support). Abbott: IDEXX (research); CEVA (program support); Boehringer Ingelheim Vetmedica (consultancy, speaking). Chetboul: Boehringer Ingelheim Vetmedica (consultancy, speaking); CEVA (speaking); Vetoquinol (consultancy, speaking); Elanco (speaking). Côté: Boehringer Ingelheim Vetmedica, Iams, IDEXX Laboratories, Nestlé Purina, Royal Canin (speaking); IDEXX Laboratories Canada, Zoetis Canada (research support). Fox: Boehringer Ingelheim Vetmedica (consultancy, speaking). Häggström: Boehringer Ingelheim Vetmedica GmbH; CEVA Sante Animale; Nestle Purina. Kittleson: none. Schober: Boehringer Ingelheim Vetmedica GmbH. Stern: Myokardia (research support); Cytokinetics (research support).

## OFF‐LABEL ANTIMICROBIAL DECLARATIONS

Authors declare no off‐label use of antimicrobials.

## INSTITUTIONAL ANIMAL CARE AND USE COMMITTEE (IACUC) OR OTHER APPROVAL DECLARATION

Authors declare no IACUC or other approval was needed.

## HUMAN ETHICS APPROVAL DECLARATION

Authors declare human ethics approval was not needed for this study.
